# Loss of OLFM4 promotes tumor migration through inducing interleukin-8 expression and predicts lymph node metastasis in early gastric cancer

**DOI:** 10.1038/oncsis.2016.42

**Published:** 2016-06-13

**Authors:** J Zhao, P Shu, F Duan, X Wang, L Min, Z Shen, Y Ruan, J Qin, Y Sun, X Qin

**Affiliations:** 1 Department of General Surgery, Zhongshan Hospital, Fudan University, Shanghai, China; 2 Department of Biochemistry and Molecular Biology, School of Basic Medical Sciences, Fudan University, Shanghai, China; 3 Institutes of Biomedical Sciences, Fudan University, Shanghai, China

## Abstract

Endoscopic surgery is increasingly used for early gastric cancer (EGC) treatment worldwide, and lymph node metastasis remains the most important risk factor for endoscopic surgery in EGC patients. Olfactomedin 4 (OLFM4) is mainly expressed in the digestive system and upregulated in several types of tumors. However, the role of OLFM4 in EGC has not been explored. We evaluated OLFM4 expression by immunohistochemical staining in 105 patients with EGC who underwent gastrectomy. The clinicopathological factors and OLFM4 expression were co-analyzed to predict lymph node metastasis in EGC. The metastatic mechanism of OLFM4 in gastric cancer was also investigated. We found that OLFM4 was upregulated in EGC tumor sections, and relatively low expression of OLFM4 was observed in patients with lymph node metastasis. OLFM4 expression as well as tumor size and differentiation were identified as independent factors, which could be co-analyzed to generate a better model for predicting lymph node metastasis in EGC patients. *In vitro* studies revealed that knockdown of OLFM4 promoted the migration of gastric cancer cells through activating the NF-κB/interleukin-8 axis. Negative correlation between OLFM4 and interleukin-8 expression was also observed in EGC tumor samples. Our study implies that OLFM4 expression is a potential predictor of lymph node metastasis in EGC, and combing OLFM4 with tumor size and differentiation could better stratify EGC patients with different risks of lymph node metastasis.

## Introduction

Though the incidence and mortality have both declined over the past several decades, gastric cancer still ranks the fifth most common malignancy and the third leading cause of cancer-related deaths worldwide.^1^ Along with the improvement of diagnostic methods and public health awareness, the number of early gastric cancer (EGC) is rapidly growing. Because of the lower complication rate, improved quality of life and similar long-term outcome compared with gastrectomy, endoscopic surgery, including endoscopic mucosal resection and endoscopic submucosal dissection, have been proposed as a replacement for conventional surgery.^2^ For more than a decade in the past, endoscopic surgery has gained increasing acceptance worldwide. As endoscopic surgery could not perform systemic lymph node sweeping, the presence of lymph node metastasis (LNM) is the most important risk factor for EGC patients. Though LNM of EGC is not common, it is still present in 3–5% of patients with mucosal cancer and 10–25% of those with submucosal cancer.^3^ Endoscopic surgery is indicated for selected patients with insignificant risk of LNM, and therefore, ruling out LNM is a crucial step before performing endoscopic mucosal resection or endoscopic submucosal dissection.^4^ Nevertheless, the diagnosis of LNM before surgery is lacking of effective and accurate methods. Morphological appearance,^5^ some clinicopathological factors^6^ and imaging technology^7^ are known to be helpful in predicting LNM but with low specificity and sensitivity. Recently, some biological markers have been found to be useful predictors, whereas the conclusion is discordant.^8, 9^

Olfactomedin 4 (OLFM4), also known as human granulocyte colony-stimulating factor-stimulated clone 1 (hGC-1), is a member of olfactomedin domain-containing protein family.^10^ The OLFM4 gene, located on chromosome 13q14.3, encoding a 510 amino acid glycoprotein, is cloned from human hematopoietic myeloid cells.^11^ OLFM4 is mainly expressed in the digestive system, such as esophagus, stomach, small intestine and colon, and is upregulated in several types of tumors, including gastric cancer.^12^ Although the biological function of OLFM4 remains unclear, recent reports suggest OLFM4 as a novel marker for the differentiation,^13^ progression^14^ and LNM in gastric cancer.^15^ However, all previous studies were focused on the relationship between OLFM4 expression and clinicopathological characteristics in all patients regardless of tumor stage, while the expression pattern of OLFM4 in EGC and its value in predicting LNM of EGC have not been explored.

The nuclear factor-kappa B (NF-κB) pathway is thought to have an important role in the process leading from inflammation to carcinogenesis.^16^ In gastric cancer, NF-κB is constitutively activated.^17^ Aberrant NF-κB activation results in tumor initiation, progression, metastasis and resistance to chemotherapy.^16^ Interleukin-8 (IL-8) is a downstream molecular of NF-κB and functions as an important tumorigenic factor within the tumor microenvironment.^18^ Our previous study also indicated a critical role of the miRNA-302c/IL-8 axis in gastric cancer metastasis.^19^ In this study, our data indicate that loss of OLFM4 promotes tumor migration through activating the NF-κB/IL-8 axis in gastric cancer cells, and identify OLFM4 expression as an independent factor which could be combined with tumor size and differentiation level to generate a better model for predicting LNM in EGC patients.

## Results

### Immunohistochemistry findings

**Figure 1:**
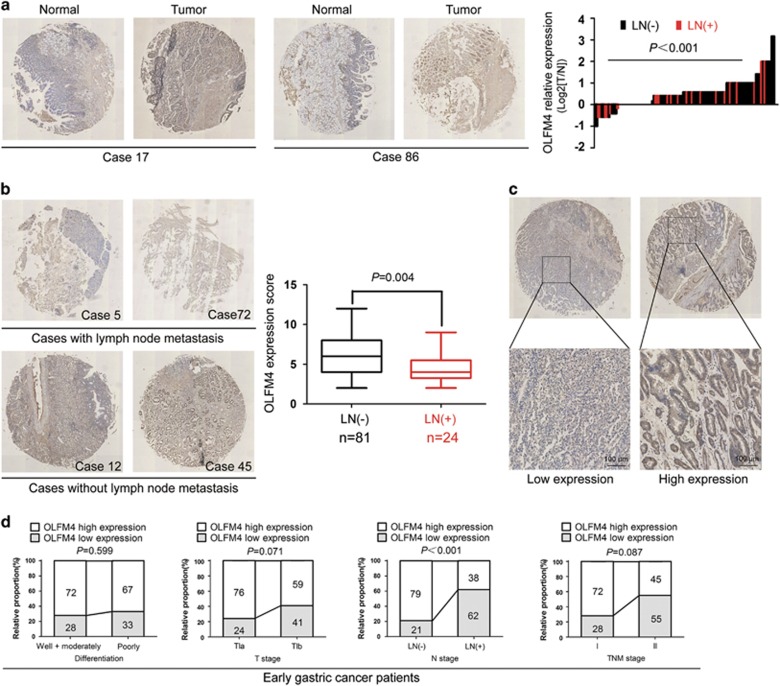
Immunohistochemical staining of the OLFM4 in sections of gastric cancer. (**a**) Representative tissue microarray of OLFM4 expression in tumor sections and adjacent non-tumor tissues. OLFM4 was highly expressed in tumor sections than non-tumor tissues (*P*<0.001). Relative OLFM4 expression was calculated as the Log2 value of T-score/N-score. N, matched non-tumor tissue. T, gastric cancer tissue; (**b**) Representative images of OLFM4 staining in tumor sections from patients with and without LNM. OLFM4 expression score was lower in patients with LNM (mean±s.e.m.: 4.63±0.44) than that in patients without LNM (mean±s.e.m.: 6.52±0.32) (*P*=0.004). The box plot shows the full range of variation (error bars: min and max) with the line representing median. (**c**) Representative microphotographs of intratumoral low and high expression of OLFM4 and their regional magnifications; Original magnification: × 200. (**d**) The percentage of patients with OLFM4 high or low expression according to the differentiation, T stage, LNM and TNM stage in EGC patients.

Tissue microarrays of 105 EGC were applied in immunohistochemistry assay to examine OLFM4 expression. Results demonstrated that OLFM4 was upregulated in EGC tumor sections compared with adjacent non-tumor tissues (*P*<0.001), and was mainly distributed within the cytoplasm of the tumor cells (Figure 1a). Moreover, we found that in EGC patients with upregulation of OLFM4, the rate of LNM was 17.98% (16/89), while in patients with OLFM4 downregulation, the rate increased to 50.00% (8/16) (Figure 1a).

### Correlations between OLFM4 expression and LNM in EGC

We next evaluated the correlations between OLFM4 expression and LNM in EGC. LNM was identified when tumor cells could be found in the lymph node. The LNM rate of EGC was 22.86% (24 out of 105) (Table 1). A total number of 2464 lymph nodes were harvested from 105 EGC patients (mean number: 23), and among them, 98 lymph nodes (metastatic lymph node ratio: 4%) were found to be metastatic from 24 patients (mean number: 4). Particularly, nearly half of EGC patients with LNM had only one to two metastatic lymph nodes. Immunohistochemical staining revealed that EGC cases with LNM commonly displayed lower OLFM4 expression compared with those without LNM (*P*=0.004) (Figure 1b). According to the receiver operating characteristic (ROC) curve, OLFM4 staining score less than 4 was considered as low expression. Representative images indicating low and high expression of OLFM4 were shown in Figure 1c. Statistical analysis demonstrated that low expression of OLFM4 was only correlated with LNM among the various clinicopathological characteristics in EGC (*P*<0.001) (Figure 1d and Table 1). However, in another group of gastric cancer cases of all stages, low expression of OLFM4 was significantly related to poor differentiation (*P*=0.011), diffuse and mixed type (*P*=0.031), higher invasion depth (*P*=0.004), LNM (*P*=0.008) as well as late TNM stage (*P*=0.002) (Supplementary Table 1). These results suggest that OLFM4 expression is specifically correlated with LNM in EGC.

### Correlations between OLFM4 expression and prognosis in gastric cancer patients

We next explored the relationship between OLFM4 expression and overall survival of gastric cancer patients in our cohort. Results demonstrated that high expression of OLFM4 in tumor tissues showed a survival advantage for gastric cancer patients at all stages. However, owing to the high survival rate of EGC patients, OLFM4 did not show prognostic value in EGC group (Supplementary Figure 1a). As our cohort lacked recurrence-free survival data, we also explored the prognostic value of OLFM4 expression by using an online survival analysis software (http://www.kmplot.com/analysis/index.php?p=service&cancer=gastric), which integrated reported microarray datasets. Similar results were observed that high expression of OLFM4 was significantly associated with better overall survival as well as progression-free survival in gastric cancer patients (Supplementary Figure 1b).

### Predictable factors for LNM in EGC

**Figure 2:**
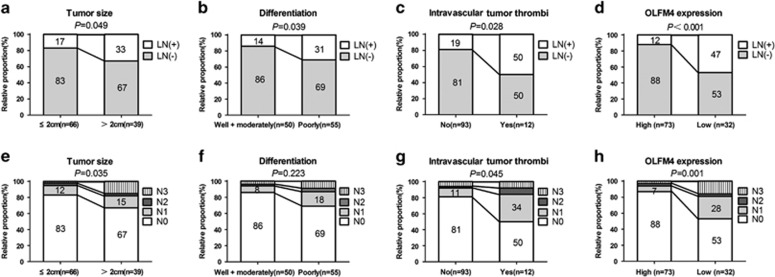
The correlation of clinicopathological features and OLFM4 expression with LNM in EGC patients. (**a**–**d**) The correlation of tumor size, differentiation, intravascular tumor thrombi and OLFM4 expression with the status of LNM in EGC patients. (**e**–**h**) The correlation of tumor size, differentiation, intravascular tumor thrombi and OLFM4 expression with the numbers of lymph nodes with metastasis in EGC patients.

We also examined the correlations between various clinicopathological features and LNM in EGC. Results demonstrated that significant correlation between LNM and larger tumor size (*P*=0.049), poorly differentiated carcinoma (*P*=0.039), intravascular tumor thrombi (*P*=0.028), as well as reduced OLFM4 expression (*P*<0.001) (Table 2 and Figures 2a–d). Moreover, in the T1a and T1b subgroups of EGC, LNM was only associated with OLFM4 expression (*P*=0.01) in T1a patients and closely related to intravascular tumor thrombi (*P*=0.042) and OLFM4 expression (*P*=0.027) in T1b patients (Table 2). In addition, OLFM4 expression (*P*=0.001) as well as tumor size (*P*=0.035) and intravascular tumor thrombi (*P*=0.045) were also associated with the number of lymph nodes with metastasis (Figures 2e–h).

### Univariate and multivariate analysis for LNM in EGC

To identify the odds ratio (OR) of clinicopathological factors for LNM in EGC, univariate analysis was conducted. Larger tumor size (OR, 2.500; 95% confidence interval (CI), 0.988–6.327), poor differentiation (OR, 2.748; 95% CI, 1.029–7.340), intravascular tumor thrombi (OR, 4.167; 95% CI, 1.202–14.442) and reduced OLFM4 expression (OR, 6.275; 95% CI, 2.345–16.791) were identified as risk factors that might affect the LNM of EGC (Table 3). Variables demonstrating a significant effect on predicting LNM were also included in the multivariate analysis. Tumor size (OR, 2.391; 95% CI, 1.057–5.407; *P*=0.036), differentiation (OR, 2.936; 95% CI, 1.033–8.343; *P*=0.043), intravascular tumor thrombi (OR, 3.826; 95% CI, 1.094–13.385; *P*=0.036) and OLFM4 expression (OR, 4.193; 95% CI, 1.859–9.458; *P*=0.001) were identified as independent predictive factors for LNM in EGC patients after adjustment of covariates (Table 3).

### ROC curve and nomogram model for predicting LNM in EGC

**Figure 3:**
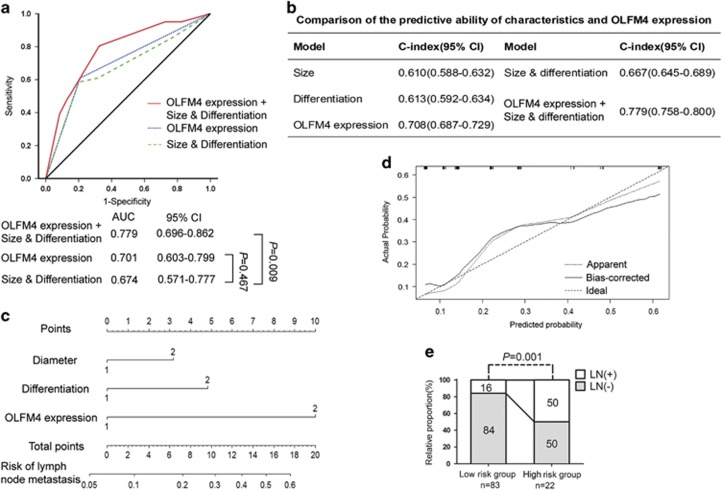
Establishment of models to predict LNM in patients with EGC. (**a**) ROC analysis for the predictive value of combined size & differentiation with OLFM4 model, size & differentiation model and OLFM4 model in LNM of EGC patients. (**b**) C-index was examined to compare the predictive accuracies of tumor size, differentiation and OLFM4 expression. (**c**) Nomogram generation for predicting LNM integrated with tumor size (1 represents<2 cm, 2 represents>2 cm), differentiation (1 represents well and moderate differentiation, 2 represents poor differentiation) and OLFM4 expression (1 represents high expression, 2 represents low expression). (**d**) Calibration curve for nomogram-predicted and observed probability of LNM. (**e**) The patients were stratified into low and high-risk groups according to nomogram-predicted score with the cutoff value determined by ROC analysis, and the percentage of patients with or without LNM in each group was demonstrated. *P*-value<0.05 was considered statistically significant.

According to the instructions published by Japan Gastric Cancer Association (JGCA) or National Cancer Centre (NCC), the traditional model to predict LNM of EGC in clinical practice is commonly established based on clinicopathological variables including tumor size and differentiation. To provide a more sensitive and specific prediction model for LNM in EGC, OLFM4 expression level was compared or combined with other clinicopathological variables demonstrating a significantly predictive value to generate the ROC curve. As vascular invasion could not be assessed by gastroscopic biopsy before surgery, it was omitted when analyzing. As shown in Figure 3a, the predictive value for LNM in EGC was comparable between OLFM4 expression (area under ROC curve (95% CI), 0.701 (0.603–0.799) and size & differentiation (area under ROC curve (95% CI), 0.674 (0.571–0.777) model (*P*=0.467), and combining OLFM4 expression with size & differentiation showed superior predictive validity (area under ROC curve (95% CI), 0.779 (0.696–0.862)) compared with traditional clinicopathological variables alone (*P*=0.009). In addition, the C-index value (95%) of OLFM4 (0.708 (0.687–0.729)) was higher than that of tumor size (0.610 (0.588–0.632)), differentiation (0.613 (0.592–0.634)) as well as size & differentiation (0.667 (0.645–0.689)). Combining with OLFM4 expression (0.779 (0.758–0.800)) generated a better predictive model than size & differentiation model alone (Figure 3b). We further constructed a nomogram that integrated the predictive factors including tumor size, differentiation and OLFM4 expression to provide a quantitative method for better predicting the LNM in EGC (Figure 3c). In the nomogram, higher total point represents higher risk of LNM. The calibration plot demonstrated that the nomogram performed well compared with the ideal prediction model (Figure 3d). We next stratified the patients with EGC into low-risk and high-risk groups according to the score calculated using the nomogram. Results demonstrated that scoring with the nomogram effectively discriminated the risk of LNM in EGC (Figure 3e). These results imply that incorporation of OLFM4 expression into clinicopathological features can establish a superior predictive model for LNM in EGC.

### OLFM4 induces the migration inhibition and morphologic change in gastric cancer cells

**Figure 4:**
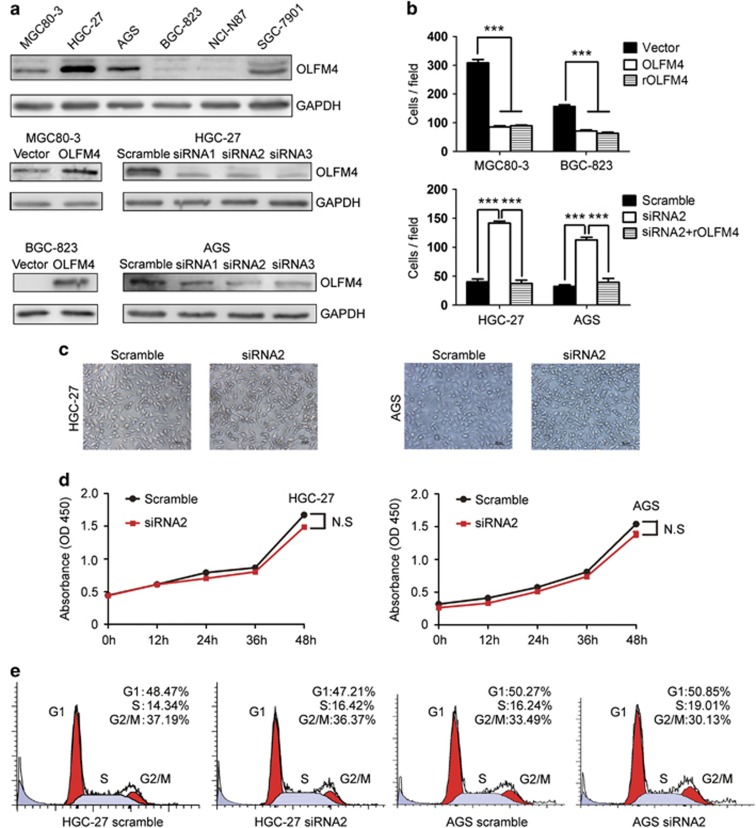
OLFM4 decreases the migration potential and induces morphologic change in gastric cancer cells. (**a**) The expression of OLFM4 in wild-type and transfected gastric cancer cells were detected by western blotting. (**b**) In the upper panel, the effects of OLFM4 overexpression or recombinant OLFM4 (100 ng/ml) on the migration of MGC80-3 and BGC-823 cells were examined. In the lower panel, the effects of OLFM4 siRNA with or without recombinant OLFM4 (100 ng/ml) on the migration of HGC-27 and AGS cells were examined with transwell assay. (**c**) The effect of OLFM4 depletion on morphologic change in gastric cancer cells. (**d**) The effects of OLFM4 siRNA on the viability of HGC-27 and AGS cells were examined with CCK-8 assay. (**e**) The effect of OLFM4 siRNA on the cell cycle of HGC-27 and AGS cells were detected by flow cytometry assay. In (**b**–**e**), experiments were repeated three times. In (**b**), error bars indicate means±s.e.m. In (**c**) and (**e**), images are representative of three independent experiments. N.S, not significant; ***P<0.001; rOLFM4, recombinant OLFM4.

We next evaluated the potential effect of OLFM4 on the migration of gastric cancer cells. As shown in Figure 4a, OLFM4 was differentially expressed in a panel of gastric cancer cell lines. HGC-27 and AGS cells with high OLFM4 expression were transfected with OLFM4-specific siRNA, while MGC80-3 and BGC-823 cells, which displayed low or faint level of OLFM4, were transfected with OLFM4 overexpression plasmids. Transwell assay demonstrated that OLFM4 overexpression attenuated the migratory potential, whereas depletion of OLFM4 enhanced cell migration *in vitro* (Figure 4b). In addition, administration of recombinant OLFM4 protein blocked cellular migration in both wild-type and OLFM4-depleted cells, implying that tumor-derived OLFM4 may inhibit the migratory activity of gastric cancer cells in an autocrine-dependent manner (Figure 4b).

As OLFM4 expression was reported to be associated with gastric cancer differentiation,^13^ we next investigated whether OLFM4 had an effect on morphology of gastric cancer cells. As shown in Figure 4c, depletion of OLFM4 resulted in visible change to a more elongated and spindled morphology. We also examined the effect of OLFM4 siRNA on the cell growth by CCK-8 assay. As shown in Figure 4d, knockdown of OLFM4 showed little effect on the viability of gastric cancer cells. Flow cytometry analysis demonstrated that depletion of OLFM4 did not lead to significant change in cell cycle distribution (Figure 4e).

### Decreased expression of OLFM4 was associated with NF-κB activation and IL-8 upregulation in gastric cancer

**Figure 5:**
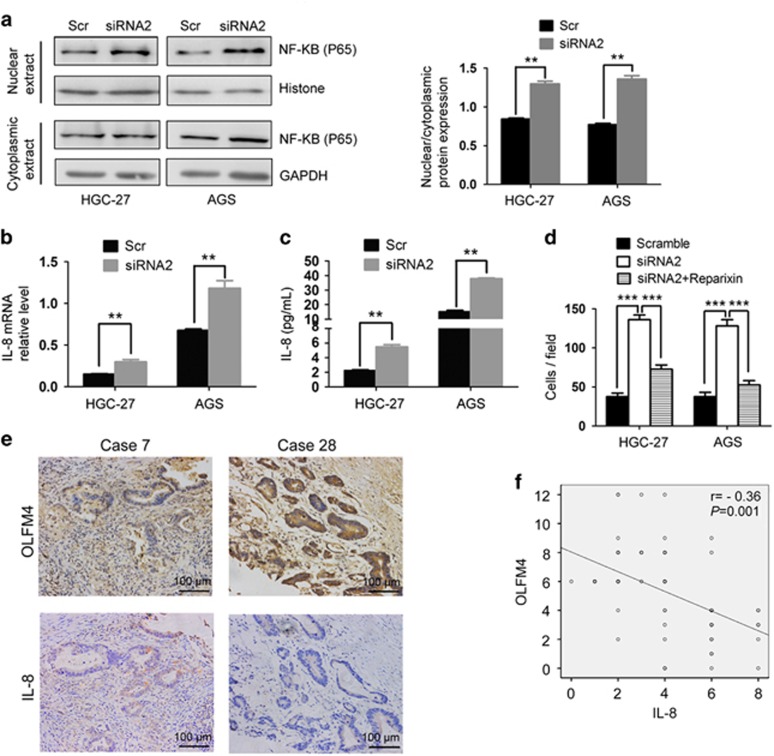
Depletion of OLFM4 induces NF-κB activation and IL-8 expression in gastric cancer. (**a**) HGC-27 and AGS cells were transfected as indicated. Forty-eight hours later, nuclear and cytoplasmic proteins were extracted and subjected to western blot analysis. (**b**) The effect of OLFM4 siRNA on IL-8 mRNA levels in HGC-27 and AGS cells were detected by real-time PCR. (**c**) The effect of OLFM4 siRNA on IL-8 protein levels in the supernatant of HGC-27 and AGS cells were detected by ELISA. (**d**) The effects of Reparixin (100 nM) on the migration of OLFM4-depleted gastric cancer cells were detected by transwell assay. (**e**, **f**) Correlation of OLFM4 with the expression of IL-8 in clinical EGC cases. Representative images in consecutive tumor sections were shown. Correlation between OLFM4 and IL-8 protein expression in 73 cases was analyzed. In (**a**–**d**), experiments were repeated three times, and error bars indicate means±s.e.m. **P<0.01; ***P<0.001; Scr, Scramble.

We next explored how OLFM4 modulated the migration of gastric cancer cells. OLFM4 has been reported to inhibit NF-κB activation via a negative feedback mechanism in the process of *Helicobacter pylori* infection.^20^ We found that in HGC-27 and AGS cells, transfection with OLFM4 siRNA induced the translocation of NF-κB subunit p65 into nucleus, suggesting the activation of NF-κB (Figure 5a). As a downstream molecule of NF-κB signaling, the expression of IL-8 was also found to be increased when OLFM4 was depleted (Figures 5b and c). We next assessed whether knockdown of OLFM4 promoted cell migration through IL-8. Results indicated that administration of Reparixin, an inhibitor of IL-8 receptor, blocked the migratory ability in OLFM4-depleted gastric cancer cells (Figure 5d). Immunohistochemical staining showed there was a reverse correlation (r=−0.36) between OLFM4 and IL-8 expression in EGC specimens (Figures 5e and f). These results suggest that decreased expression of OLFM4 is associated with NF-κB activation and IL-8 upregulation in gastric cancer.

## Discussion

Overall incidence of gastric cancer has steadily declined over decades, particularly in developed countries. In contrast, the percentage of cases diagnosed at the stage of ‘EGC' has greatly increased. The frequency of EGC in Western countries is not really known owing to lack of screening programs for this tumor entity.^21^ In Eastern countries, particularly in Japan and South Korea, EGC accounts for almost 50% of all gastric cancer cases.^22^ For years, surgical treatment is the gold standard for EGC. However, because the endoscopic mucosal resection or endoscopic submucosal dissection is introduced to endoscopic therapy, they are widely used in Eastern countries, especially in Japan, South Korea and also in China.^23^ The absolute indications for endoscopic therapy based on the Japan Gastric Cancer Association (JGCA) is differentiated, non-ulcerated, clinically T1a (limited within mucosa) tumor of 2 cm or less in diameter.^24^ On the other hand, the expanded National Cancer Centre (NCC) criteria recommend that the differentiated mucosal cancer regardless of size in the absence of ulceration or less than 3 cm in diameter in the presence of ulceration can be endoscopically managed.^25^ LNM is a major problem related to tumor recurrence and limit the wide application of endoscopic therapy in EGC patients.

Therefore, assessing the existence of LNM is the crucial issue before the performance of endoscopic surgery. Both JGCA and NCC guidelines are based on the clinicopathological investigations of gastroscopic biopsies. In addition, imaging technology is also applied to evaluate the existence of LNM preoperatively. Computed tomography scan is the most widely used method for preoperative staging; however, single LNM is commonly observed in EGC with LNM, and the sensitivity and specificity is about 50% in this situation.^7^ Endoscopic ultrasonography is another important modality for predicting LNM. Nevertheless, the predictive accuracy is only 55–75%^26^ because it is deeply influenced by the size of the lymph node. Genetic or protein markers for predicting LNM in EGC patients are being gradually recognized. Several biological markers, such as E-cadherin, vascular endothelial growth factor and matrix metalloprotease,^27, 28, 29, 30^ are found to be associated with LNM, however, no protein marker has been added to establish a predicting model and their clinical value has not been proved so far.

The expression of OLFMF4 is tissue-specific, and has shown to have crucial roles in the development and progression of digestive cancers. Liu *et al.*^31^ demonstrated knockdown of OLFM4 gene inhibited cell growth by regulating cell cycle progression in gastric cancer. However, in our study, we found the viability and cell cycle were not obviously changed in OLFM4-depleted cells. The correlation between OLFM4 expression and tumor size was also not observed in immunohistochemical staining. However, we found that OLFM4 showed an obvious inhibitory effect on migration of gastric cancer cells, which was consistent with a previous report.^32^ In addition, Guo *et al.*^33^ also demonstrated that silencing of OLFM4 could enhance gastric cancer cell invasion. These results indicate that OLFM4 may have a more important role in regulating metastasis rather than proliferation of gastric cancer. We also found that depletion of OLFM4 induced a more elongated and spindled morphologic change in gastric cancer cells, which was supported by our and other's observations that OLFM4 was correlated with gastric cancer differentiation.^13^ In addition, the underlying mechanism how OLFM4 modulates the differentiation of cancer cells may need further investigation.

Liu *et al.*^20^ previously indicated that OLFM4 had a negative feedback effect on NF-κB activation in the *H.* *pylori*-infected gastric mucosa, and OLFM4 suppressed NF-κB activation possibly through a direct association with nucleotide oligomerization domain-1 (NOD1) and -2 (NOD2). In our study, we also verified the negative correlation between OLFM4 and NF-κB activation in transformed gastric cancer cells, and found that NF-κB/IL-8 pathway was critical for OLFM4 depletion-induced migration of cancer cells. OLFM4 expression was also significantly reversely correlated with IL-8 expression in gastric cancer tissues. It has been well recognized that IL-8 constitutes an example of a cytokine released by tumor cells that simultaneously function in an autocrine and paracrine mode within the tumor microenvironment.^18^ Therefore, loss of OLFM4 may help in building a niche to drive the progression of gastric cancer.

Previous clinical correlation analysis revealed that reduction of OLFM4 expression was associated with poor differentiation, late tumor stage and poor survival,^14, 15^ which was consistent with our findings. However, all previous studies were focused on the clinical significance of OLFM4 in all gastric cancer patients regardless of tumor stage, whereas the expression of OLFM4 in EGC and its potential value in predicting LNM of EGC have not been evaluated. Our data demonstrate that OLFM4 is upregulated in EGC, and specifically correlated with LNM in EGC patients. Though OLFM4, as well as traditional clinicopathological variables, including size and differentiation, have been identified as independent predictable factors, we find that OLFM4 is the only factor correlated with LNM in both T1a and T1b subgroups, suggesting the clinical importance of OLFM4 in assessing LNM in EGC patients (Table 2). Next, a model combining the OLFM4 expression with size & differentiation features is generated to evaluate the risk of LNM in EGC patients, and has been proven to be effective in discriminating low-risk group that may be suitable for endoscopic surgery in our patient samples (Figure 3).

In conclusion, our data suggest OLFM4 as a new biomarker to establish the risk model for predicting LNM of EGC. In addition, because loss of OLFM4 expression in EGC is associated with the activation of NF-κB/IL-8 axis, targeting NF-κB/IL-8 would be of potential benefit for preventing LNM in EGC cases with low OLFM4 expression.

## Materials and methods

### Patients and gastric cancer specimens

A total of 105 EGC patients and another group of 102 gastric cancer cases of all stages were recruited in this study. All of the patients had undergone standard gastrectomy with regional lymph node resection from Zhongshan Hospital (Shanghai, China) between 1999 and 2008. None of them received any preoperative treatment. The specimens were all resected by pathologists. The patients' characteristics including age, gender, tumor size, location, differentiation, Lauren classification, intravascular thrombi and TNM stage were obtained from medical records and reassessed independently by two pathologists and physicians according to the 2010 American Joint Committee on Cancer TNM classification system. The use of human tissue samples and clinical data was approved by the ethics committee of Fudan University and was performed in accordance with the ethical standards laid down in the 1964 Declaration of Helsinki and its later amendments. All donors were informed of the aim of the study and gave consent to donate their samples.

### Tissue arrays and immunohistochemistry

Two core tissue biopsies (1.5 mm in the greatest dimension) were taken from the center of each formalin-fixed and paraffin-embedded gastric tumor foci and arranged on glass slides in sequence. In brief, slides were baked at 60° for 6 h, followed by deparaffinization with xylene, rehydrating in graded ethanol and blocking the endogenous peroxidase activity in 3% hydrogen peroxide. UltraVision Protein Block (Thermo Scientific, Fremont, CA, USA) was then applied to block nonspecific background staining. The sections were submerged in citrate buffer and microwaved for antigen retrieval. Sections were then incubated with the primary antibody at 4 °C. After washing, tissue sections were treated with Primary Antibody Amplifier Quanto and HRP Polymer Quanto (Thermo Scientific). Then DAB Quanto (Thermo Scientific) was applied to tissue sections. Finally, tissue sections were counterstained with hematoxylin, dehydrated and covered with coverslips for further analysis. The slides were scanned by the use of a computerized image system composed of an Olympus CCD camera (Tokyo, Japan) connected to a Nikon eclipse Ti-s microscope (Tokyo, Japan) and captured by NIS-Elements F3.2. All of the slides were assessed by two gastroenterology pathologists who had no knowledge of the patients' clinical data to exclude subjectivity. The immunohistochemistry was evaluated by the percentage and the intensity of staining cells. The percentage of immunopositive stained cells (A) was divided into five grades as: <10% (score 0); 10–29% (score 1); 30–49% (score 2); 50–69% (score 3); and >70% (score 4). The intensity of staining (B) was categorizes as: negative (0), weak (1), moderate (2) and strong (3). The total score for each section was measured as A × B,^34^ and the cutoff score was determined by the ROC analysis. The value on the curve closest to the point (0, 1) which maximized both sensitivity and specificity for the LNM was considered to be the cutoff score.^35^

### Cell lines, antibodies and reagents

The human gastric cancer cell lines AGS, HGC-27, BGC-823 and MGC80-3 were purchased from the Cell Bank of the Type Culture Collection of the Chinese Academy of Sciences (Shanghai, China), and cultured in DMEM or RPMI-1640 supplemented with 10% fetal bovine serum (Gibco, Grand Island, NY, USA) at 37 °C in a humidified atmosphere containing 5% CO2. The primary antibodies against OLFM4 (ab85046), IL-8 (AF-208) and p65 (8242S) were purchased from Abcam (New Territories, Hong Kong), R&D Systems (Minneapolis, MN, USA) and Cell Signaling Technology (Beverly, MA, USA), respectively. The recombinant human OLFM4 protein (11639-H08H-10) was purchased from Sino Biological Inc (Beijing, China). The inhibitor of CXCL8 receptor, Reparixin (HY-15251), was purchased from MedChem Express (Monmouth Junction, NJ, USA). The Nuclear and Cytoplasmic Protein Extraction Kit (P0027) was purchased from Beyotime Institute of Biotechnology (Nantong, China).

### Plasmid construction and RNA interference

The OLFM4 plasmid was synthesized by Shanghai Genechem Co., LTD (Shanghai, China). The OLFM4 control small interfering RNA (siRNA) was designed by Biotend Research (Shanghai, China) with the following sequences: GAGGUGGAGAUAAGAAAUA-dTdT (siRNA1), GGGCAAACUAGACAUUGUA-dTdT (siRNA2) and GGAGACUGUUGGAGUAUUA-dTdT (siRNA3). Lipofectamine 2000 (Life Technologies, Carlsbad, CA, USA) was used for transfection according to the instructions.

### Western blotting

Cell lysates were separated by sodium dodecyl sulfate–polyacrylamide gel electrophoresis, transferred onto polyvinylidene difluoride membranes and incubated with primary antibodies, followed by incubation with horseradish peroxidase-conjugated secondary antibodies. Protein expression was visualized by enhanced chemiluminescence assay.

### RNA extraction and real-time PCR

Briefly, total RNA of cancer cells was isolated according to the manufacturer's protocol (Life Technologies) and the cDNA was generated by the Takara RNA PCR Kit. Real-time PCR was performed with SYBR Premix Ex Taq (Takara, Otsu, Japan) agent in StepOne Plus (Life Technologies). The primers were as follows: OLFM4 forward, GACCAAGCTGAAAGAGTGTGAGG; OLFM4 reverse, CCTCTCCAGTTGAGCTGAACCA; IL-8 forward, GAATGGGTTTGCTAGAATGTGATA; IL-8 reverse, CAGACTAGGGTTGCCAGATTTAAC; GAPDH forward, GCCGGTGCTGAGTATGTC; and GAPDH reverse, CTTCTGGGTGGCAGTGAT.

### Migration assays

*In vitro* cell migration assays were conducted by using transwell chambers (8 μm pore size; Millipore, Billerica, MA, USA). Approximately 3 × 10^4^ gastric cells were suspended in 200 μl of serum-free medium and seeded into the upper chamber. The lower chamber was filled with 600 μl of complete growth medium. After 24 h, the infiltrating cells were stained with crystal violet, and five fields were observed for counting cell numbers.

### Enzyme-linked immunosorbent assay

HGC-27 and AGS cells were cultured in six-well plates. After transfection with OLFM4 siRNA for 48 h, the supernatants were collected and measured for IL-8 expression by ELISA kits according to the manufacturer's instructions (D8000C, R&D Systems, Minneapolis, MN, USA).

### Cell proliferation assay

Briefly, HGC-27 and AGS cells were transfected as indicated. Twenty-four hours later, transfected cells were seeded in 96-well plates. WST-8 dye (Beyotime Institute of Biotechnology) was used to incubate cells for the indicated times. Absorbance was determined at 450 nm with a Universal Microplate Reader (Bio-Tek Instruments, Winooski, VT, USA).

### Cell cycle analysis

Briefly, after indicated treatment, cells were collected by trypsin, washed twice with phosphate-buffered saline, and then were stained with DNA staining solution and propidium iodide for 30 min by using Cell Cycle Staining Kit (Lianke Bio, Hangzhou, China). Stained cells were assessed by flow cytometer and data were analyzed by FlowJo software (TreeStar, Ashland, OR, USA).

### Statistical analysis

Statistical analysis was performed with SPSS (Version 18, Chicago, IL, USA) and R (http://www.r-project.org/) software. Correlations between clinical variables, OLFM4 expression and LNM were analyzed by using Pearson's test or Fisher's exact test. Binary Logistic regression was used to perform on multivariate analysis of independent predictable factors for LNM. The Kaplan–Meier method was used to determine survival probability and differences were assessed by the log-rank test. ROC analysis was used to compare the sensitivity and specificity for the prediction of LNM by the parameters. The Harrell's concordance index (C-index) was used to judge the predictive accuracy of the models. A nomogram was created with R using the ‘rms' package to be a new prediction model. A calibration plot was generated to examine the performance characteristics of the nomogram. All statistical analyses were two-sided, and *P*<0.05 was considered statistically significant.

## Figures and Tables

**Table 1:** Relation between intratumoral OLFM4 expression and clinical characteristics of early gastric cancer

*Factor*	*Patients*		*OLFM4 expression*		
	*No.*	*%*	*Low*	*High*	P*-value*
All patients	105	100	32	73	
Age (years)					0.603
⩽65	65	61.9	21	44	
>65	40	38.1	11	29	
Gender					0.846
Female	51	48.6	16	35	
Male	54	51.4	16	38	
Localization					0.374
Proximal	6	5.7	2	4	
Middle	33	31.4	7	26	
Distal	66	62.9	23	43	
Diameter					0.071
⩽2cm	66	62.8	16	50	
>2cm	39	37.2	16	23	
Differentiation					0.599
Well+Moderately	50	47.6	14	36	
Poorly	55	52.4	18	37	
Lauren classification					0.134
Intestinal type	70	66.7	18	52	
Diffuse type+Mixed type	35	33.3	14	21	
Intravascular tumor thrombi					0.175
No	93	88.6	25	68	
Yes	12	11.4	6	6	
T classification					0.071
T1a	66	62.9	16	50	
T1b	39	37.1	16	23	
N classification					<**0.001**
LN (−)	81	77.1	17	64	
LN (+)	24	22.9	15	9	
TNM stage					0.087
I	94	89.5	26	68	
II	11	10.5	6	5	

Abbreviations: LN, lymph node; TNM, tumor node metastasis. P*-value*<0.05 marked in bold font shows statistical significance.

**Table 2:** Relation between lymph node metastasis and clinical and pathological characteristics of early gastric cancer

*Factors*	*All T1 patients*					*T1a*					*T1b*				
	*Patients*		*Lymph node metastasis*			*Patients*		*Lymph node metastasis*			*Patients*		*Lymph node metastasis*		
	*No.*	*%*	*(*−*)*	*(+)*	P*-value*	*No.*	*%*	*(*−*)*	*(+)*	P*-value*	*No.*	*%*	*(*−*)*	*(+)*	P*-value*
All patients	105	100	81	24		66	100	53	13		39	100	28	11	
Age					0.065					0.319					0.243
⩽65	65	61.9	54	11		45	68.2	38	7		20	51.3	16	4	
>65	40	38.1	27	13		21	31.8	15	6		19	48.7	12	7	
Gender					0.276					0.668					0.477
Female	51	48.6	37	14		27	40.9	21	6		24	61.5	16	8	
Male	54	51.4	44	10		39	59.1	32	7		15	38.5	12	3	
Localization					0.357					0.345					1
Middle+Proximal	39	37.1	32	7		24	36.4	21	3		15	38.5	11	4	
Distal	66	62.9	49	17		42	63.6	32	10		24	61.5	17	7	
Diameter					**0.049**					0.105					0.482
⩽ 2 cm	66	62.8	55	11		44	66.7	38	6		22	56.4	17	5	
>2 cm	39	37.2	26	13		22	33.3	15	7		17	43.6	11	6	
Differentiation					**0.039**					0.095					0.471
Well+Moderately	50	47.6	43	7		34	51.5	30	4		16	41	13	3	
Poorly	55	52.4	38	17		32	48.5	23	9		23	59	15	8	
Intravascular tumor thrombi					**0.028**					0.337					**0.042**
No	93	88.6	75	18		60	90.9	49	11		33	84.6	26	7	
Yes	12	11.4	6	6		6	9.1	4	2		6	15.4	2	4	
Lauren classification					0.622					0.731					0.767
Intestinal type	70	66.7	53	17		43	65.2	34	9		27	69.2	19	8	
Diffuse+Mixed type	35	33.3	28	7		23	34.8	19	4		12	30.8	9	3	
OLFM4 expression					<**0.001**					**0.01**					**0.027**
Low	32	30.5	17	15		16	24.2	9	7		16	41	8	8	
High	73	69.5	64	9		50	75.8	44	6		23	59	20	3	

Abbreviations: TNM, tumor node metastasis; T1a, tumor does not reach submucosa; T1b, tumor infiltrates to submucosa. *P*-value<0.05 marked in bold font shows statistical significance

**Table 3:** Univariate and multivariate logistic regression analyses for lymph node metastasis of early gastric cancer

*Factor*	*Univariate*		*Multivariate*	
	*OR (95% CI)*	P*-value*	*OR (95% CI)*	P*-value*
Age		0.065		
⩽65	1.00 (reference)			
>65	2.364 (0.936–5.969)			
Gender		0.276		
Male	1.00 (reference)			
Female	1.665 (0.662–4.185)			
Localization		0.357		
Middle+proximal	1.00 (reference)			
Distal	1.586 (0.591–4.253)			
Diameter		**0.049**		**0.036**
⩽2 cm	1.00 (reference)		1.00 (reference)	
>2 cm	2.5 (0.988–6.327)		2.391 (1.057–5.407)	
Differentiation		**0.039**		**0.043**
Moderately+well	1.00 (reference)		1.00 (reference)	
Poorly	2.748 (1.029–7.340)		2.936 (1.033–8.343)	
Intravascular tumor thrombi		**0.028**		**0.036**
No	1.00 (reference)		1.00 (reference)	
Yes	4.167 (1.202–14.442)		3.826 (1.094–13.385)	
Lauren classification		0.622		
Intestinal	1.00 (reference)			
Diffuse+mixed	1.283 (0.476–3.460)			
OLFM4 expression		**< 0.001**		**0.001**
High	1.00 (reference)		1.00 (reference)	
Low	6.275 (2.345–16.791)		4.193 (1.859–9.458)	

Abbreviations: 95% CI, 95% confidence interval; OR, odds ratio. *P*-value<0.05 marked in bold font shows statistical significance.
