# Supplementary figures and images for: Loss of OLFM4 promotes tumor migration through inducing interleukin-8 expression and predicts lymph node metastasis in early gastric cancer
Source: Oncogenesis. 2016 Jun 13;5(6):e234–. doi: 10.1038/oncsis.2016.42 (PMC4945743; doi:10.1038/oncsis.2016.42)

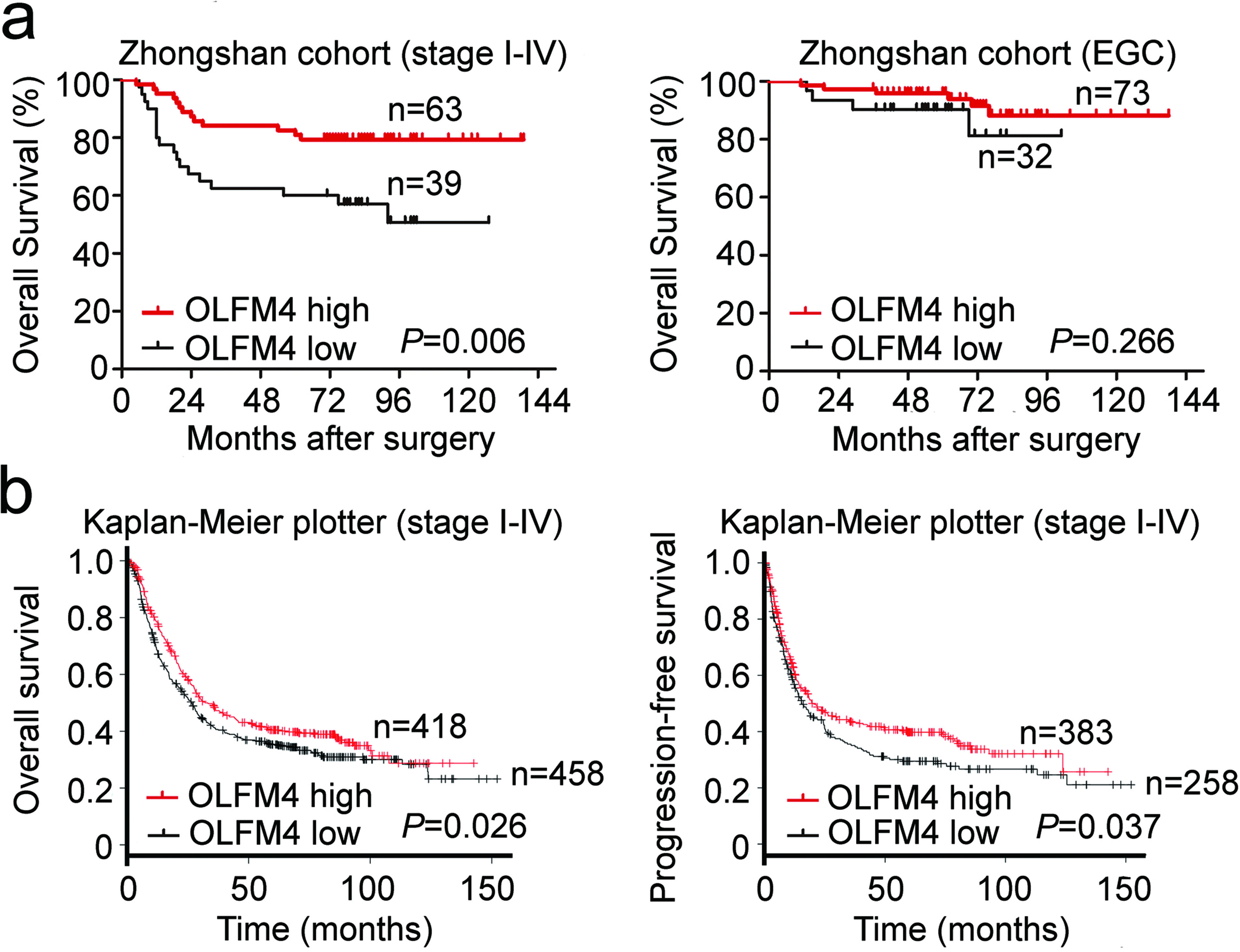

Supplement: Supplementary Figure [file oncsis201642x1.tif]
